# Detection of genetic variation in *Ocimum* species using RAPD and ISSR markers

**DOI:** 10.1007/s13205-014-0269-y

**Published:** 2014-12-09

**Authors:** Hardik K. Patel, Ranbir S. Fougat, Sushil Kumar, Jigar G. Mistry, Mukesh Kumar

**Affiliations:** Department of Agricultural Biotechnology, Anand Agricultural University, Anand, 388 110 India

**Keywords:** Basil, Diversity, ISSR, *Ocimum*, RAPD, Tulsi

## Abstract

There is a lack of information on the molecular characterization of *Ocimum* species and hence, efforts have been made under the present study to characterize 17 *Ocimum* genotypes belonging to 5 different species (*O. basilicum, O. americanum, O. sanctum, O. gratissimum* and *O. Polystachyon*) through random amplified polymorphic DNA (RAPD) and inter simple sequence repeats (ISSR) markers. PCR amplification using 20 RAPD primers generated a total of 506 loci, of which 490 (96.47 %) loci were found polymorphic. The PIC value for RAPD ranged from 0.907 (OPF 14) to 0.954 (OPC 11) with an average of 0.937. The ISSR primers generated a total of 238 loci, of them 234 (98.17 %) loci were polymorphic. The PIC value ranged from 0.892 (UBC 808) to 0.943 (ISSR A12) with an average of 0.923. The average Jaccard’s similarity coefficient based on RAPD and ISSR analysis was 0.58 and 0.52, respectively. Clustering pattern of dendrogram generated using the pooled RAPD and ISSR data showed all *Ocimum* genotypes in their respective species groups at a cutoff value of 0.49 and 0.42, respectively. Many unique species-specific alleles were amplified by RAPD and ISSR markers. In both marker systems, a maximum number of unique alleles were observed in *O. sanctum*. The results of the present investigation provided valid guidelines for collection, conservation and characterization of *Ocimum* genetic resources.

## Introduction

Interest in the exploitation of medicinal and aromatic plants as pharmaceuticals, herbal remedies, flavorings, perfumes and cosmetics, and other natural products has greatly increased in the recent years (Anonymous 1994; Ayensu 1996). India is an innate emporium of many medicinal plants and most of such plants are used traditionally. *Ocimum* like other medicinal plants are highly valued medicinal plant in the traditional Ayurvedic and Unani system of medicine for its range of therapeutic activities. It belongs to the family Lamiaceae, which has close to 252 genera and 6,700 species (Mabberley 1997), most of which are used for medicinal purpose (Wren 1968) and find diverse uses in the indigenous system of medicine in many countries like Africa, Saudi Arabia, Australia, Burma, India, Malaya, Pacific Islands and Sri Lanka (Pushpangadan and Sobti 1977; Balyan and Pushpangadan 1988). Many species of this genus are also used as pot herbs.

*O. sanctum* L. (Tulsi), *O. gratissimum* (Ram Tulsi, 2*n* = 40), *O. canum* (Dulal Tulsi; 2*n* = 24), *O. basilicum* (Ban Tulsi), *O. polystachyon* (2*n* = 60), *O. americanum* and *O. micranthum* are examples of known important species of genus *Ocimum* which nurture in different parts of the world. Plants have square stems, fragrant opposite leaves and whorled flower on spiked inflorescence. Basic chromosome number in *Ocimum* species is *x* = 12 (Carovic et al. 2010), whereas, *O. basilicum* and *O. americanum* are reported to be tetraploid (2*n* = 4*x* = 48) and hexaploid (2*n* = 6*x* = 72), respectively (Sobti and Pushpangadan 1979). *O. sanctum* is perennial shrub with a basic chromosome number of *n* = 8 (Darrah 1980; Pushpangadan and Bradu 1995).

Important essential oil constituents reported from *Ocimum* species include linalool, linalyl acetate, geraniol, citral, camphor, eugenol, methyl eugenol, methyl chavicol, methyl cinnamate, thymol, safrole etc., which are of immense value in the perfumery and cosmetic industries (Balyan and Pushpangadan 1988) and also shown to have antibacterial activity. Among *Ocimum* species, common basil viz. *O. basilicum* is economically the most important one. The aromatic leaves of basil are used fresh and dried as flavoring agents or spices in a wide variety of foods. Volatile oils of basil are used to flavor foods, dental and oral products, and in fragrances. Basil is also used in traditional ceremonial rituals. It also contains biologically active constituents that are insecticidal, nematicidal, fungistatic, or antimicrobial. *O. sanctum*’ possesses antifertility, anticancer, antidiabetic, antifungal, hepatoprotective, cardioprotective, antiemetic, antispasmodic, analgesic and antitussive properties (Singh et al. 2011). The two main morphotypes of *O. sanctum* cultivated in India are (1) green-leaved plants known as Sri Tulsi (Green Tulsi) and (2) purple-leaved plants known as Krishna Tulsi (Black Tulsi) (Raina et al. 2013).

Because of its potential uses as a traditional medicine, incorporation of *Ocimum* species into agro forestry systems would not only make the species accessible to the majority of the rural population that uses it but also contribute to its genetic conservation. However, before this programme of widespread domestication of the species is implemented, it would be important to determine its genetic diversity so that only elite genotypes are multiplied and conserved (Harisaranraj et al. 2008).

Molecular markers have proven to be powerful tools in the assessment of genetic variation and in the elucidation of genetic relationships within and among species. Random amplified polymorphic DNA (RAPD) markers have been used to characterize the genetic diversity in a number of medicinal and aromatic plants including *Ocimum* (Satovic et al. 2002; Vieira et al. 2003; Singh et al. 2004; De Masi et al. 2006). The advantage of RAPDs is that they require no prior sequence information (Palumbi 1996). Inter simple sequence repeats (ISSR) technique is also a PCR-based method, which involves amplification of DNA segment present at an amplifiable distance in between two identical microsatellite repeat regions oriented in opposite direction. It is a reproducible, highly polymorphic marker and is useful in studies of genetic diversity, phylogeny, gene tagging, genome mapping and evolutionary biology (Reddy et al. 2002). There is a lack of information on the molecular characterization of the *Ocimum* species. To accomplish the above aim, the present investigation was carried out with RAPD and ISSR.

## Materials and methods

### Plant material and DNA extraction

Seeds of 17 genotypes belonging to 5 species were procured from AICRP on Medicinal and Aromatic Plants, Anand Agricultural University, Anand (Table 1). Ten plants of each accession were grown in pots for DNA isolation. Two grams of young leaf tissue was harvested from each plant and frozen in liquid nitrogen for DNA extraction. DNA from young leaves of a bulk of ten plants was isolated using CTAB technique (Doyle and Doyle 1987), purified and quantified using Nanodrop N.D.1000 (Software V.3.3.0, Thermo Scientific, USA). DNA was diluted to 20 ng/μl with T_10_E_1_ buffer and stored at 4 °C.

**Table 1:** Details of genotypes used for RAPD and ISSR based characterization

Sr. no.	Genotypes	Species name
1	Green Tulsi	*O. sanctum*
2	Black Tulsi	
3	Kapur Tulsi	
4	Closimum	*O. gratissimum*
5	Van Tulsi	
6	Aajlo	*O. americanum*
7	Aavachi Bavachi	*O. polystachyon*
8	SBOB-1	*O. basilicum*
9	SBOB-2	
10	Walmi	
11	Violet	
12	Jodhpur	
13	Jhadol Udaipur	
14	Solan Serrated	
15	IC-283658	
16	Long Spike	
17	Anand Local	

### RAPD and ISSR amplification

A total of 120 primers (100 RAPD and 20 ISSR) were used in PCR amplification. RAPD primers used in this study were selected from the study of Singh et al. (2004), while ISSR primers of UBC series were selected from the report of Aghaei et al. (2012). PCR amplification was carried out using 200 µl PCR tubes (Axygen, USA) in thermocyclers (Biometra, Germany). PCR amplification was carried out in a 25 μl reaction volume containing 2.5 μl template DNA (50 ng), 1× Dream Taq PCR buffer with MgCl_2_ (Fermentas, USA), 0.4 μl (5 U/μl) Taq polymerase (Fermentas, USA), 0.5 μl (2.5 mM each) dNTPs (Fermentas, USA) and 1 μl (10 pmol/μl) primer (MWG Biotech, Germany). RAPD-PCR was performed at an initial denaturation at 94 °C for 5 min, 38 cycles of 94 °C for 1 min, 38 °C for 1 min, 72 °C for 1.2 min, and final extension at 72 °C for 5 min. The optimal annealing temperature for ISSR primers was found to vary according to the base composition of the primers. Therefore, ISSR-PCR was performed at an initial denaturation temperature of 94 °C for 5 min, 38 cycles of 94 °C for 50 s, 35–58 °C (depending on primer sequence) for 60 s and 72 °C for 1.2 min and a final extension of 72 °C for 10 min.

### Agarose gel electrophoresis

Amplified products were electrophoresed in 1.5 % agarose in 1× TBE buffer. The gels were stained with ethidium bromide and documented using gel documentation system (Bio-Rad, Hercules, CA, USA). Each experiment was repeated two times with each primer and those primers which gave reproducible fingerprints (DNA bands) were only considered for the data analysis.

### Data analysis

For each genotype, each fragment/band that was amplified using ISSR and RAPD primers was treated as unit character. Unequivocally reproducible bands were scored and entered into a binary character matrix (1 for presence and 0 for absence). The pairwise genetic similarity coefficient was calculated using Jaccard’s coefficient (Jaccard 1908) by the SIMQUAL program of NTSYS-pc software version 2.02 (Rohlf 1998). A dendrogram was constructed based on the matrix of distance using unweighted pair group method with arithmetic averages (UPGMA).

To compare the efficiency of primers, polymorphic information content (PIC), as a marker discrimination power, was computed using the formula PIC = $1 - \sum {P_{i}^{2} }$, where *P*_*i*_ is the frequency of the *i*th allele at a given locus (Anderson et al. 1993). The PIC values are commonly used in genetics as a measure of polymorphism for a marker locus using linkage analysis. Correlation between the matrices obtained with both marker types (RAPD and ISSR primers) was estimated by means of Mantel test using MxComp module of NTSYSpc (Mantel 1967). Principal component analysis was carried out using the EIGEN module of NTSYSpc 2.02.

## Results

### Performance of different marker systems

#### ISSR analysis

**Fig. 1:**
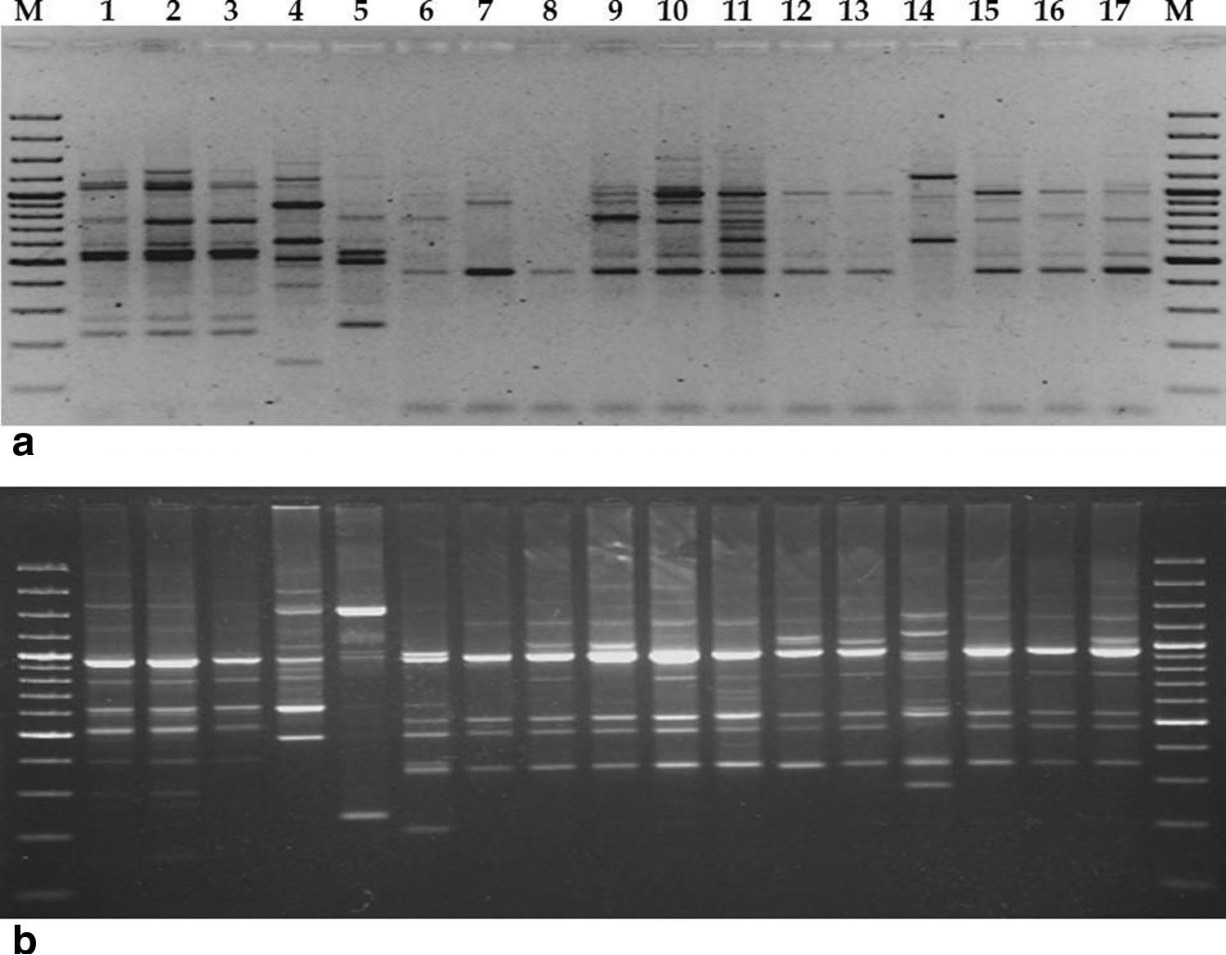
**a** ISSR profile 17 *Ocimum* genotypes generated by UBC 807 and **b** RAPD profile of 17 *Ocimum* genotypes generated by OPD 10

Among 20 ISSR primers used in this study, 12 primers detected a total of 238 amplicons in 17 genotypes, out of which 234 (98.17 %) were polymorphic (Fig. 1a; Table 2). Out of 12 primers, eight primers were 100 % polymorphic. The number of total amplicons varied from 13 (UBC 808) to 32 (ISSR A12) with an average of 19.5 loci per primer, and sizes ranged from 89 (UBC 807) to 2,940 bp (UBC 841). The number of polymorphic amplicons ranged from 12 (UBC 808) to 31 (ISSR A12) with an average of 19.5 polymorphic loci per marker. The PIC value ranged from 0.892 (UBC 808) to 0.943 (ISSR A12) with a mean of 0.923. Marker index value for ISSR was 17.99. ISSRs were also highly efficient with respect to molecular species identification.

**Table 2:** Numerical data as obtained from PCR amplification by ISSR primers among *Ocimum* species

Sr. no.	Locus name	Sequence 5′–3′	GC content (%)	Tm	Molecular weight (bp)	No. of loci	No. of polymorphic loci	Percent polymorphism (%)	PIC value
1	UBC 443	ACACACACACACACACACT	47	49	453–2,159	18	17	94.44	0.909
2	UBC 807	AGAGAGAGAGAGAGAGT	47	45	89–1,556	22	22	100	0.930
3	UBC 808	AGAGAGAGAGAGAGAG(CT)C	53	51	604–2,224	13	12	92.30	0.892
4	UBC 818	CACACACACACACAG	53	42	342–1,835	18	17	94.44	0.915
5	UBC 825	ACACACACACACACACT	47	45	446–2,279	20	20	100	0.934
6	UBC 834	AGAGAGAGAGAGAGAG(CT)T	47	49	392–2,597	19	19	100	0.934
7	UBC 840	GAGAGAGAGAGAGAGAYT	47	45	222–1,952	18	18	100	0.919
8	UBC 841	GAGAGAGAGAGAGAGAYC	53	47	189–2,940	22	22	100	0.937
9	UBC 855	ACACACACACACACACYT	47	45	238–2,284	15	15	100	0.908
10	ISS RA7	AGAGAGAGAGAGAGAGAGAGT	48	52	254–1,720	21	21	100	0.930
11	ISS RA12	GAGAGAGAGAGACC	57	40	225–1,984	32	31	96.88	0.943
12	CTC 4RC	CTCCTCCTCCTCRC	69	44	307–2,587	20	20	100	0.922
Total					–	238	234	–	–
Average					313–176	19.8	19.5	98.17	0.923

#### RAPD analysis

In present study, 17 accessions belonging to 5 different basil species were surveyed with two marker systems, i.e., RAPD and ISSR. For RAPD analysis, among 100 arbitrary primers tested, 20 primers generated 506 loci (Fig. 1b; Table 3). Of these, 490 loci were polymorphic with an average polymorphism of 96.47 %. Out of 20 primers, nine primers were 100 % polymorphic. The molecular size of the amplified RAPD products ranged from 152 bp (OPC 18) to 3,176 bp (OPF 5). The number of total amplicons varied from 16 (OPA 3) to 33 (OPC 11) with an average of 25.3 loci per primer. The number of polymorphic amplicons ranged from 15 (OPA 2 and OPA 3) to 33 (OPC 11) with an average of 24.5 loci per primer. OPC 11 showed the highest PIC (0.954), while it was lowest for OPF 14 (0.907) with an average of 0.937. Marker index value for RAPD was 22.95.

**Table 3:** Numerical data as obtained from PCR amplification by RAPD primers among *Ocimum* species

Sr. no	Locus name	Sequence 5′–3′	GC content (%)	Molecular weight (bp)	No. of loci	No. of polymorphic loci	Polymorphism (%)	PIC value
1	OPA 2	TGCCGAGCTG	70	362–2,312	17	15	88.23	0.93
2	OPA 3	AGTCAGCCAC	60	333–1,607	16	15	93.75	0.92
3	OPA 4	AATCGGGCTG	60	189–2,746	23	19	82.61	0.94
4	OPA 9	GGGTAACGCC	70	266–2,923	19	18	94.74	0.92
5	OPA 11	CAATCGCCGT	60	206–2,518	22	21	95.45	0.94
6	OPC 4	CCGCATCTAC	60	236–2,717	30	29	96.67	0.94
7	OPC 11	AAAGCTGCGG	60	214–2,812	33	33	100	0.95
8	OPC 14	TGCGTGCTTG	60	237–3,073	31	30	96.77	0.95
9	OPC 15	GACGGATCAG	60	602–2,625	32	32	100	0.94
10	OPC 18	TGAGTGGGTG	60	152–2,067	30	30	100	0.94
11	OPD 2	GGACCCAACC	70	319–2,566	27	27	100	0.93
12	OPD 3	GTCGCCGTCA	70	248–2,795	29	29	100	0.94
13	OPD 10	GGTCTACACC	60	176–1,912	27	26	96.30	0.93
14	OPD 11	AGCGCCATTG	60	204–2,256	25	25	100	0.94
15	OPD 18	GAGAGCCAAC	60	373–1,919	24	23	95.83	0.92
16	OPD 20	ACCCGGTCAC	70	266–2,270	28	28	100	0.95
17	OPE 9	CTTCACCCGA	60	187–2,801	34	32	94.12	0.94
18	OPF 5	CCGAATTCCC	60	285–3,176	22	22	100	0.93
19	OPF 8	GGGATATCGG	60	269–2,013	20	19	95	0.92
20	OPF 14	GGTCTAGAGG	60	336–2,112	17	17	100	0.91
Total				–	506	490	–	–
Average				176–3,176	25.3	24.5	96.47	0.937

Using ISSR and RAPD for *Ocimum* DNA analysis, species-specific DNA fragments were identified. ISSR and RAPD amplicons occurring only within a given species and showing no polymorphism at the intra-specific level were considered to be species-specific markers. A total of four ISSR primers produced species-specific amplicons with maximum of three amplicons in *O. sanctum*. However, no species-specific alleles were detected in *O. basilicum* (Table 4). Similarly, out of the 20 RAPD primers analyzed, three primers produced species-specific amplicons (Table 4). The primer OPF 8 generated two amplicons (1,180 bp and 269 bp), specific to *O. sanctum* and *O. gratissimum*, respectively. *O. americanum* and *O. polystachyon* were not considered in RAPD- and ISSR-based species-specific allele detection as both species were represented by only single genotype each.

**Table 4:** *Ocimum* species-specific bands as revealed by RAPD and ISSR markers

*Ocimum* species	Characterized by the RAPD markers (bp)	Characterized by the ISSR markers (bp)
*O. Sanctum*	OPD 18–439	UBC 808–520
	OPF 8–1,180	UBC 825–450
		UBC 840–1,110
*O. gratissimum*	OPF 8–269	CTC 4RC–1,180
*O. basilicum*	OPC 4–1,370	–

#### RAPD-based cluster analysis

Jaccard’s similarity coefficients based on RAPD markers among the all pair-wise combinations of genotypes ranged from 0.21 [between Van Tulsi (*O. sanctum*) and Aavachi Bavachi (*O. polystachyon*)] to 0.90 (between Long Spike and Anand Local) with an average value of 0.39. *O. basilicum* group showed a high genetic similarity index as compared to other species (Table 5). Genetic similarity within the *O. basilicum* genotypes varied from 0.36 (SBOB-1 and Solan Serrated) to 0.90 (Long Spike and Anand Local), and average genetic similarity coefficient was 0.58.

**Table 5:** Jaccard’s similarity coefficient based on RAPD analysis in 17 *Ocimum* genotypes

Genotypes	Green Tulsi	Black Tulsi	Kapur Tulsi	Closimum	Van Tulsi	Aajlo	Aavachi Bavachi	SBOB-1	SBOB-2	Walmi	Violet	Jodhpur	Jhadol Udaipur	Solan Serrated	IC-283658	Long Spike	Anand Local
Green Tulsi	1.00																
Black Tulsi	0.88	1.00															
Kapur Tulsi	0.75	0.78	1.00														
Closimum	0.31	0.31	0.32	1.00													
Van Tulsi	0.24	0.24	0.23	0.44	1.00												
Aajlo	0.27	0.26	0.25	0.23	0.33	1.00											
Aavachi Bavachi	0.22	0.23	0.23	0.22	0.21	0.38	1.00										
SBOB-1	0.23	0.24	0.22	0.23	0.24	0.36	0.43	1.00									
SBOB-2	0.26	0.26	0.24	0.23	0.24	0.38	0.43	0.62	1.00								
Walmi	0.27	0.27	0.24	0.26	0.26	0.38	0.34	0.50	0.65	1.00							
Violet	0.25	0.26	0.22	0.25	0.24	0.37	0.34	0.49	0.57	0.70	1.00						
Jodhpur	0.23	0.24	0.22	0.21	0.22	0.35	0.37	0.53	0.62	0.62	0.63	1.00					
Jhadol Udaipur	0.24	0.25	0.22	0.23	0.24	0.40	0.37	0.53	0.64	0.65	0.60	0.78	1.00				
Solan Serrated	0.31	0.32	0.27	0.26	0.25	0.37	0.25	0.36	0.39	0.40	0.43	0.42	0.46	1.00			
IC-283658	0.26	0.27	0.24	0.25	0.25	0.39	0.35	0.48	0.56	0.57	0.55	0.64	0.70	0.49	1.00		
Long Spike	0.25	0.25	0.23	0.23	0.23	0.38	0.39	0.56	0.60	0.61	0.59	0.70	0.75	0.43	0.82	1.00	
Anand Local	0.25	0.26	0.23	0.23	0.22	0.38	0.38	0.55	0.61	0.62	0.59	0.69	0.75	0.42	0.77	0.90	1.00

**Fig. 2:**
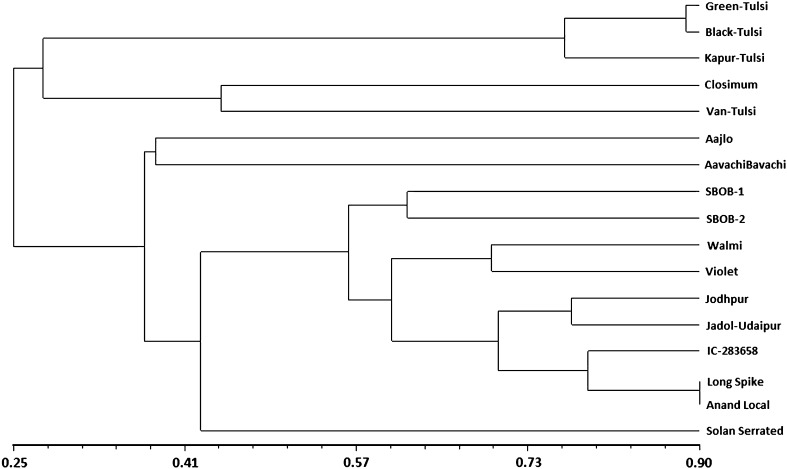
UPGMA cluster analysis of 17 *Ocimum* genotypes with Jaccard’s similarity coefficient of RAPD

The UPGMA clustering algorithm based on RAPD data grouped 17 accessions into six groups at an average cutoff value of 0.49 (Fig. 2). The cophenetic correlation was calculated and indicated a very good fit (*r* = 0.98). RAPD clearly distinguished all the species. Among the clusters, most of the samples from *O. basilicum* were clustered into group two (9 samples) than the other, while group seven contained three genotypes all belonging to *O. sanctum*. However, a genotype namely Solan serrated was found quite distinct from the other cultivars of *O. basilicum* as it clustered apart from the group two. Remaining groups contained only a single sample each. Two genotypes Closimum and Van Tulsi belonging to *O. gratissimum* were grouped as different clusters.

#### ISSR-based cluster analysis

The values of similarity coefficient obtained in ISSR analysis ranged from 0.13 (between Closimum and Aavachi) to 0.96 (between Green Tulsi and Black Tulsi) among the genotypes studied (Table 6). The average similarity coefficient among genotypes was 0.35. Within the *O. basilicum* genotypes, genetic similarity ranged from 0.29 (SBOB-1 and Solan Serrated) to 0.84 (Long Spike and Anand Local) with an average genetic similarity coefficient of 0.52.

**Table 6:** Jaccard’s similarity coefficient based on ISSR analysis in 17 *Ocimum* genotypes

Genotypes	Green Tulsi	Black Tulsi	Kapur Tulsi	Closimum	Van Tulsi	Aajlo	Aavachi Bavachi	SBOB-1	SBOB-2	Walmi	Violet	Jodhpur	Jhadol Udaipur	Solan Serrated	IC-283658	Long Spike	Anand Local
Green Tulsi	1.00																
Black Tulsi	0.96	1.00															
Kapur Tulsi	0.84	0.86	1.00														
Closimum	0.24	0.24	0.25	1.00													
Van Tulsi	0.20	0.19	0.20	0.48	1.00												
Aajlo	0.18	0.18	0.19	0.21	0.31	1.00											
Aavachi Bavachi	0.18	0.18	0.19	0.13	0.20	0.41	1.00										
SBOB-1	0.17	0.17	0.17	0.22	0.23	0.44	0.44	1.00									
SBOB-2	0.16	0.16	0.17	0.17	0.23	0.45	0.48	0.59	1.00								
Walmi	0.19	0.19	0.18	0.25	0.27	0.44	0.40	0.56	0.65	1.00							
Violet	0.21	0.21	0.20	0.27	0.30	0.39	0.37	0.51	0.49	0.71	1.00						
Jodhpur	0.18	0.17	0.18	0.18	0.26	0.44	0.44	0.51	0.68	0.60	0.49	1.00					
Jhadol Udaipur	0.16	0.16	0.15	0.22	0.26	0.37	0.33	0.43	0.56	0.60	0.51	0.64	1.00				
Solan Serrated	0.24	0.24	0.21	0.25	0.33	0.34	0.26	0.28	0.30	0.35	0.36	0.33	0.32	1.00			
IC-283658	0.23	0.23	0.22	0.22	0.30	0.35	0.30	0.33	0.35	0.38	0.37	0.37	0.36	0.72	1.00		
Long Spike	0.20	0.19	0.20	0.19	0.23	0.41	0.37	0.46	0.54	0.56	0.51	0.63	0.51	0.33	0.47	1.00	
Anand Local	0.21	0.21	0.21	0.22	0.25	0.40	0.35	0.47	0.56	0.57	0.51	0.60	0.54	0.36	0.45	0.84	1.00

**Fig. 3:**
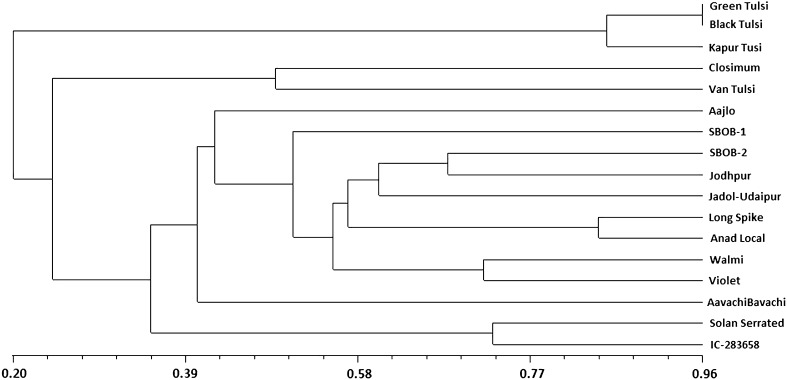
UPGMA cluster analysis of 17 *Ocimum* genotypes with Jaccard’s similarity coefficient of ISSR

Based on the genetic similarities, seventeen genotypes were grouped into six major clusters at a cutoff value of 0.42 (Fig. 3) and the cophenetic correlation showed a good fit (*r* = 0.97). The clustering of genotypes proved the suitability of ISSRs in detecting alleles characteristic of genotypes from different species. Group two contained only one genotype of *O. polystachyon* that accommodating itself in *O. basilicum* group, consequently dividing the *O. basilicum* into two clusters (1 and 3). The cluster one consisted of two genotypes while cluster 3 was composed of eight cultivars from *O. basilicum*. Unlike RAPD, both genotypes of *O. gratissimum* grouped in one cluster. In contrast to RAPD, *O. polystachyon* was found closer to *O. basilicum* instead of *O americanum* in ISSR analysis. Solan serrated and IC-283658 belonging to *O. basilicum* species were more diverse among all *O. basilicum* genotypes.

### RAPD- and ISSR-based principal component analysis (PCA)

**Fig. 4:**
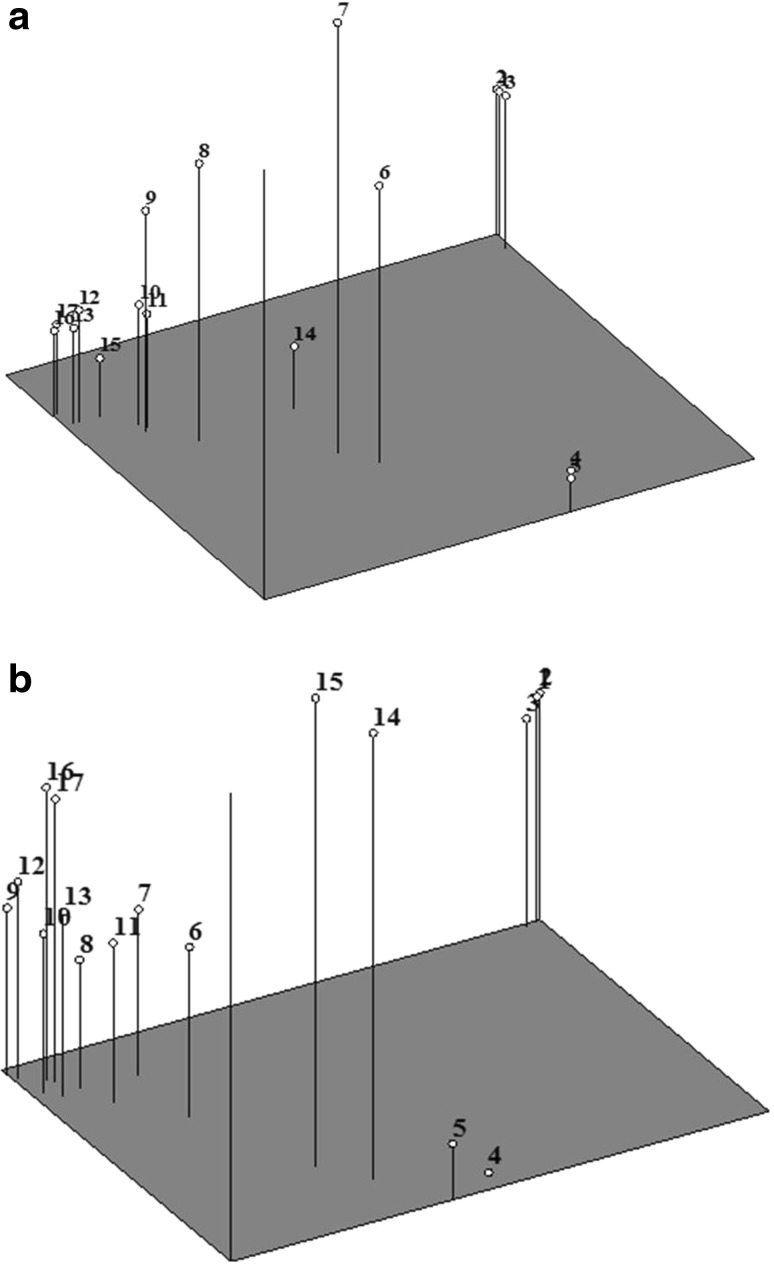
Three-dimensional plot by PCA using **a** RAPD primers and **b** ISSR primers; *1* Green Tulsi, *2* Black Tulsi, *3* Kapur Tulsi, *4* Closimum, *5* Van Tulsi, *6* Aajlo, *7* Aavachi Bavachi, *8* SBOB-1, *9* SBOB-2, *10* Walmi, *11* Violet, *12* Jodhpur, *13* Jhadol Udaipur, *14* Solan Serrated, *15* IC-283658, *16* Long Spike, *17* Anand Local

The result of PCA based on RAPD markers was comparable to the cluster analysis (Fig. 4a). The first three coordinate axis accounted for 65 % (first axis = 31 %) of the variation observed. The RAPD-based PCA revealed that the genotypes belonging to a particular cluster were grouped together in the PCA plot. It showed that the *O. sanctum* genotypes clustered together, whereas *O. basilicum* genotypes clustered as a second group. Solan serrated belonging to *O. basilicum* species was more diverse among the all *O. basilicum* genotypes. PCA analysis with ISSR markers revealed that the first three coordinate axes of analysis accounted for 63 % (first axis = 28 %) variation, and clustered all of the varieties in four clades (Fig. 4b). These results are consistent with those obtained with the ISSR dendrogram, indicating substantial genetic diversity among genotypes. It is also evident from genotypic data of both markers’ system that genotypes are fairly dispersed on PCA plots, which reflects a good genetic base. Mantel test-based correlation of similarity matrices generated by individual marker systems was 0.86.

## Discussion

Little published information can be found about assessment of molecular diversity in *Ocimum* using a PCR-based approach (Lal et al. 2012; Chen et al. 2013). The intrinsic genetic diversity in present study on *Ocimum* accessions was apparent from the analysis of their RAPD and ISSR profiles and from the dendrogram generated where all the accessions had unambiguously separated from each other. RAPD and ISSR studies have been widely used for population genetic studies in both wild (Dikshit et al. 2007, Yao et al. 2008) and cultivated plants (Nagaoka and Ogihara 1997; Sikdar et al. 2010). Generally, all these studies have reported that ISSR primers produce more reliable and reproducible bands than RAPD primers. In the present study, however, it was observed that once the PCR conditions are well set, high reproducibility for both RAPD and ISSR markers can be obtained. In general, all 22 markers used in this study produced clear consistent and reproducible amplification profiles.

### Performance of different marker systems

The plants of *Ocimum* species are valued as spice and herbal medicine in India (Harisaranraj et al. 2008). Driven by commercial incentives, the wild populations of these species have been threatened with depletion in recent years due to excessive harvesting. In the present study, 17 *Ocimum* accessions belonging to 5 species were studied with 2 different marker systems, i.e., RAPD and ISSR for genetic diversity analysis. ISSR showed the highest polymorphism level (98.17 %) than RAPD (96.47 %). The results were consistent with genetic diversity analysis of *Ocimum* by Chen et al. 2013. Similarly, Lal et al. (2012) carried out ISSR analysis in six species of *Ocimum* and found 100 % polymorphism. High polymorphism of ISSR and RAPD markers was also reported in many previous studies, for examples, RAPD of *Melocanna baccifera* (Lalhruaitluanga and Prasad 2009), ISSR of *Jatropha curcas* accessions (Grativol et al. 2011). Moreover, Dutta et al. (2010) checked the efficiency of three PCR-based markers (ISSR, RAPD and SSR) in chickpea and pigeon pea and reported higher polymorphism in all three markers. High levels of polymorphism found in the present work showed that both markers are suitable for genetic diversity studies and are equally effective to differentiate the closely related cultivars of *Ocimum*.

PIC analysis can be used to evaluate markers so that the most appropriate marker can be selected for genetic mapping and phylogenetic analysis (Anderson et al. 1993; Powell et al. 1996). Though, RAPDs cover the whole genome for amplification, ISSR amplifies the regions between two microsatellites, but the average PIC of both marker systems was higher and almost comparable. The higher PIC value for the RAPD obtained makes its MI (measure of the efficiency to detect polymorphism) much higher than that of the ISSRs used in the present study. This was consistent with previous reports in *Ocimum* (Lal et al. 2012). The high MI is the reflection of efficiency of marker to simultaneously analyze a large number of bands, rather than level of polymorphism detected (Powell et al. 1996).

Both the DNA marker analysis methods allowed us to identify species specific markers. In both marker systems, the highest number of species-specific loci was revealed in *O. sanctum*. The results of this study support that both marker types are powerful tools in resolving species/inter-species status of *Ocimum* and in deciding the distinctness of different genera within a family and species-specific alleles can be converted into co-dominate SCAR markers for further characterization of the *Ocimum* species from the different geographical regions.

### Genetic diversity analysis of basil accessions

Comparison of genetic similarity coefficients of both RAPD and ISSR markers showed that the former ranged from 0.21 to 0.90, while the latter varied from 0.13 to 0.96. Thus, both RAPD and ISSR markers showed polymorphisms and large variability; and distinguishing genotypes clearly. High level of genetic dissimilarity among *Ocimum* species demonstrates that the level of genetic variation in the species is substantial and indicated that genetic base is quite broad. However, the wide range of similarity (0.13–0.96) was observed in ISSR analysis indicating that higher genetic variations existed in the target genome regions than those targeted by RAPD (0.21–0.90). In the present study, genetic distance values were well correlated between marker types. Comparison of different marker systems for diversity in *Ocimum* revealed congruent diversity estimates for different types of markers (Chen et al. 2013). Furthermore, six groups were obtained using ISSR or RAPD. This was consistent with the higher correlation (*r* = 0.86) of the ISSR and RAPD similarity matrices and their cophenetic values. High correlation values between two marker systems have been reported earlier in many plants species (Kesari et al. 2010; Yildiz et al. 2011). In both the UPGMA-based dendrograms, the genotypes under the same species were clustered together. For RAPD and ISSR markers, a high reproducibility in dendrogram topologies was obtained, with some differences in ISSR where *O. polystachyon* shuffled between *O. basilicum* genotypes. Both markers aim to amplify a different region of the genome, and thus it is reasonable that there are some fine differences between the two dendrograms based on an individual data set. In previous reports, RAPD and ISSR also showed some differences in the positioning of few individuals (Kesari et al. 2010). In ISSR-based dendrogram, almost all accessions belonging to *O. basilicum* were clustered in Group 3, except two accessions, Solan serrated and IC-283658, indicating that these two accessions are more distantly related to the other *O. basilicum* accessions analyzed and probably may be because of some unique repeat sequences. In the present investigation, the mean similarity index of ISSR was 0.35 which is quit low as compared to the mean similarity index reported by Aghaei et al. (2012) where mean similarity index was 0.735 with same 12 ISSR primers on 50 genotypes of *O. basilicum*. Difference in number of species studied and region of collection may be probable reason for the difference in the similarity index. Lal et al. (2012) also reported a low mean similarity index (0.39).

Principal coordinate analysis was used to illustrate the multiple dimensions of the distribution of the genotypes in a scatter-plot. Separation of individual accessions to their respective clusters as is evident from the UPGMA dendrogram as well as PCA was observed for both the markers with some differences. This multivariate approach was used to complement the information obtained from the cluster analysis methods because it is more informative regarding distances among major groups (Taran et al. 2005).

## Conclusion

It can be concluded from the present study that both RAPD and ISSR produced many unique alleles which can be converted in SCAR to develop species-specific diagnostic markers.
